# Prevalence and serotype distribution of nasopharyngeal carriage of *Streptococcus pneumoniae* in China: a meta-analysis

**DOI:** 10.1186/s12879-017-2816-8

**Published:** 2017-12-13

**Authors:** Lin Wang, Jinjian Fu, Zhuoxin Liang, Jichang Chen

**Affiliations:** 1 Department of Science and Education, Liuzhou Municipal Maternity and Child Healthcare Hospital, Liuzhou, Guangxi China; 2 Department of Laboratory, Liuzhou Municipal Maternity and Child Healthcare Hospital, Liuzhou, Guangxi China; 3 Department of Pediatrics, Liuzhou Municipal Maternity and Child Healthcare Hospital, Liuzhou, Guangxi China; 4 Department of Neonatology, Liuzhou Municipal Maternity and Child Healthcare Hospital, Liuzhou, Guangxi China

**Keywords:** *Streptococcus Pneumoniae*, Healthy children, Serotype distribution, Meta-analysis

## Abstract

**Background:**

To explore the overall prevalence and serotype distribution of nasopharyngeal carriage of *Streptococcus pneumoniae*(*S. pneumoniae*) among healthy children.

**Methods:**

A search for pneumococcal nasopharyngeal carriage studies including children published up to July 31th, 2016 was conducted to describe carriage in China. The review also describes antibiotic resistance in and serotypes of *S. pneumoniae* and assesses the impact of vaccination on carriage in this region. Summary measures for overall prevalence, antibiotic resistance, and serotype distributions extracted from the analyzed data were determined with 95% confidence intervals (CIs) using random-effects models. Heterogeneity was assessed using *I* ^*2*^ test statistics.

**Results:**

Thirty-seven studies were included in this review, and the majority of studies (64.9%) were located in the pre-introduction period of 7-valent pneumococcal conjugate vaccine (PCV7) in China. The pooled prevalence of *S. pneumoniae* nasopharyngeal carriage was 21.4% (95% CI: 18.3–24.4%). Carriage was highest in children attending kindergartens [24.5%, (19.7–29.3%)] and decreased with increasing age. Before the introduction of PCV7 into China, the prevalence of *S. pneumoniae* nasopharyngeal carriage was 25.8% (20.7–30.9%), the pooled carriage of *S. pneumoniae* sharply dropped into the 14.1% (11.3–16.9%) by PCV7 vaccination period (*P* < 0.001). Before the pneumococcal conjugate vaccine (PCV) was introduced in China, the penicillin resistance rate in *S. pneumoniae* isolated from healthy children was 31.9% (21.2–42.6%); however, this rate sharply decreased after the introduction of PCV7 in China [21.6%, (7.4–35.9%)], and the difference between the rates during these two time periods was statistically significant (*P* value <0.05). Serotypes 19F, 6A and 23F were the most commonly isolated. Meta-analysis of data from young children showed a pooled rate estimate of 46.6% (38.8–54.4%) for PCV7 vaccine coverage and 66.2% (58.6–73.8%) for PCV13 vaccine coverage.

**Conclusions:**

The prevalence of nasopharyngeal carriage among children was high in China. PCV7 immunization was found to be associated with reduction of nasopharyngeal colonization of *S. pneumoniae*. Conjugate vaccination coverage was slightly affected by the introduction of PCV7 into China because of low vaccination rate. The government should implement timely adjusted conjugate vaccination strategies based on our findings.

**Electronic supplementary material:**

The online version of this article (10.1186/s12879-017-2816-8) contains supplementary material, which is available to authorized users.

## Background

*Streptococcus pneumoniae* (*S. pneumoniae*) is a major pathogen that can cause invasive pneumococcal disease (IPD) and respiratory tract infections and result in high morbidity and mortality. The World Health Organization has reported that nearly 500,000 children under 5 years of age are infected by *S. pneumoniae* annually, and the vast majority of these infections occur in developing countries [1]. Asymptomatic nasopharyngeal carriage of *S. pneumoniae* is an essential element of the transmission of pneumococcal disease [2], a prerequisite for the occurrence of invasive pneumococcal disease, and a known risk factor for subsequent acute and recurrent otitis media [3, 4].

The prevalence of nasopharyngeal pneumococcal carriage has been found to vary in different countries and regions [5]. Because *S. pneumoniae* carriage is more common than the *S. pneumoniae* disease, it is important to investigate carriage status to evaluate the effect of new pneumococcal vaccines [6]. When the 7-valent pneumococcal vaccine was introduced in mainland China, the invasive pneumococcal disease burden decreased sharply, especially disease caused by the vaccine type (VT) serotypes; this decrease was accompanied by an increase in non-vaccine type (NVT) serotype, particularly serotype 19A, as previously seen in Europe [7, 8].

This systematic review was conducted to describe the nasopharyngeal carriage status of *S. pneumoniae* in healthy children, describe the major serotypes of *S. pneumoniae*, and evaluate the impact of pneumococcal vaccination on the coverage of PCV7.

## Methods

### Literature search

The following databases were searched for relevant articles through July 31, 2016 without language limitations: PubMed, Web of Science, EMBASE, CNKI, and WANFANG database. Keywords used for this search were: (“China” OR “Chinese”), (“nasal” OR “nasopharyngeal” OR “oropharyngeal”), (“children” OR “pediatric” OR “paediatric”), (“carriage” OR “colonization” OR “colonisation”) “*Streptococcus pneumoniae*”, “serotypes”, “pneumococcal vaccine”.

### Inclusion and exclusion criteria

Studies were required to meet the following criteria for inclusion in this meta-analysis: (1) subjects were healthy children, (2) samples were collected from nasopharyngeal or oropharyngeal swabs, (3) studies focused on non-vaccination group and (4) sufficient information was provided to compute positive carriage rates and their 95% confidence intervals (CIs). Exclusion criteria were as follows: (1) if a study included both adults and children, only children data were enrolled, (2) studies reporting clinical infectious diseases caused by *S. pneumoniae*, (3) if studies included both vaccinated and non-vaccinated children, only non-vaccinated data were enrolled, (4)studies with a lack of sufficient baseline information to compute carriage rates and their 95CIs, (5) review studies, or conference studies or newspaper articles, (6) studies determining antibiotic resistance rates without carriage data, or studies were referred to infections rather than colonization, and (7) duplicate reports.

### Data extraction

Two reviewers (LW and JF) independently identified and extracted the following data: first authors, sample year, study location, study population, number of participants, number of participants with pneumococcal carriage, pre/post vaccination period, vaccination history, type of swabs, immediately incubated into plates or not, transportation period, culture plates, culture into the 5% CO_2_ or not, identification methods, serotyping methods, storage medium, rates of antibiotic resistance, and prevalence of *S. pneumoniae* serotypes and their corresponding 95% CIs.

### Quality assessment

The quality of included studies was assessed in accordance with the STROBE statement [9], studies with scores <8 were excluded from the systematic review.

### Statistical analysis

STATA version 10.0 was used to perform the statistical analyses. DerSimonian and Laird random-effects models (REM) were used to pool the data. Funnel plots were used to examine publication bias, which was further assessed using Egger’s test, with *P* < 0.10 indicating potential bias [10]. Stratified analyses were carried out to assess the heterogeneity across subgroup defined by age and PCV7 vaccination period.

## Results

### Characteristic of included studies

**Fig. 1:**
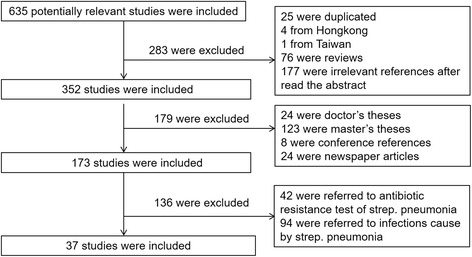
Flow chart of the study selection process

The flow chart in Fig. 1 depicts the selection process for the included studies. Overall, 614 studies were written in Chinese, and 21 studies were written in English. By reviewing the titles and abstracts, 487 articles were excluded; by using the inclusion/exclusion criteria, 37 articles were selected for further investigation that included a total of 18,881 children. They were all cross-sectional studies. The main characteristics of the studies are listed in Table 1. The first study of nasopharyngeal carriage of *S. pneumoniae* in healthy children was conducted in two kindergartens in Beijing in 1999. All samples were from nasopharyngeal and nasal swabs. The ages of the healthy children included in the studies ranged from 0 to 14 years.

**Table 1:** The characteristic of the included studies

Author	Sample year	Location	Population	Pre/post vaccination period	Vaccination history	Number of participants	Number of participants with pneumococcal carriage	Quality scores
Guoling Ping [11]	2009	Beijing	12–18 months	Post	No	600	47	17
Yakun Liu [12]	2005	Hubei	kindergarten	Pre	No	297	78	12
Yan Kang [13]	2010	Heilongjiang	kindergarten	Post	N/A	100	23	13
Liping Zhang [14]	2011	Donguan	12–18 months	Post	No	600	115	14
Hongmei Yang [15]	2011	Hubei	kindergarten & > 5 years	Post	N/A	301	66	14
Fan Yang [16]	1997–1998	Shanghai	kindergarten	Pre	No	791	222	14
Yali Liu [17]	2009	National	12–18 months	Post	No	3635	451	15
Hao Li [18]	2000	Heinan	kindergarten	Pre	No	571	151	12
Xiyuan Zhao [19]	2005	Zhongshan	>5 years	Pre	No	327	25	11
Ancun Hou [20]	1995–2000	Beijing	All age groups	Pre	No	307	57	16
Jun Liu [21]	2005	Shenyang	kindergarten	Pre	No	110	14	11
Fuqin Li [22]	2005	Hebei	kindergarten	Pre	No	100	24	12
Jianping Liang [23]	2003	Guangdong	kindergarten	Pre	No	186	61	12
Mingzhi Di [24]	2010	Beijing	All age groups	Post	1.8%vaccinated	221	45	17
Yongming He [25]	2005	Guangdong	kindergarten	Pre	No	350	121	12
Chunzhen Hua [26]	2004	Zhejiang	kindergarten	Pre	No	1220	67	14
Sangjie Yu [27]	2000	Beijing	kindergarten	Pre	No	502	190	19
Ziyong Sun [28]	2007	Wuhan	kindergarten	Pre	No	605	135	16
Hong Zhou [29]	2002	Guangdong	kindergarten	Pre	No	150	35	15
Lihua Zhang [30]	2005	Guangdong	kindergarten	Pre	No	344	132	13
Hui Wang [31]	1999	Beijing	kindergarten	Pre	No	985	244	16
Hui Chen [32]	2010	Guangdong	kindergarten	Post	N/A	120	16	15
Jing Zhang [33]	2004	Wuhan	kindergarten	Pre	No	469	116	14
Aiying Bai [34]	2010	Shandong	12–18 months	Post	No	611	57	16
Zhipeng Gao [35]	2012	Beijing	kindergarten	Post	Half vaccinated	472	103	18
Benquan Wu [36]	2000	Guangdong	kindergarten	Pre	No	220	53	17
Lihua Jiang [37]	2014	Guangxi	kindergarten & > 5 years	Post	N/A	1475	148	18
Zhigang Lai [38]	2006	Guangdong	kindergarten	Pre	No	344	132	15
Defeng Zhao [39]	2009	Wuhan	12–18 months	Post	No	596	75	18
Youqun Zeng [40]	2003	Chongqing	All age groups	Pre	No	400	76	12
NY Lee [41]	1998–1999	Beijing	kindergarten	Pre	No	267	100	17
Jiayu Hu [42]	2009	Shanghai	12–18 months	Post	No	614	102	18
Xiaoming Luo [43]	2002	Guangdong	All age groups	Pre	No	199	60	12
Yanhui Liu [44]	2006	Guangdong	kindergarten	Pre	No	400	138	11
Yanjie Liu [45]	2007	Liaoning	kindergarten	Pre	No	130	17	10
Xinghua Cao [46]	2012	Heilongjiang	kindergarten	Post	N/A	345	9	12
Dongke Chen [47]	1999	Beijing	kindergarten	Pre	No	156	56	12

### Nasopharyngeal carriage rates of *S. pneumoniae* in healthy children

**Fig. 2:**
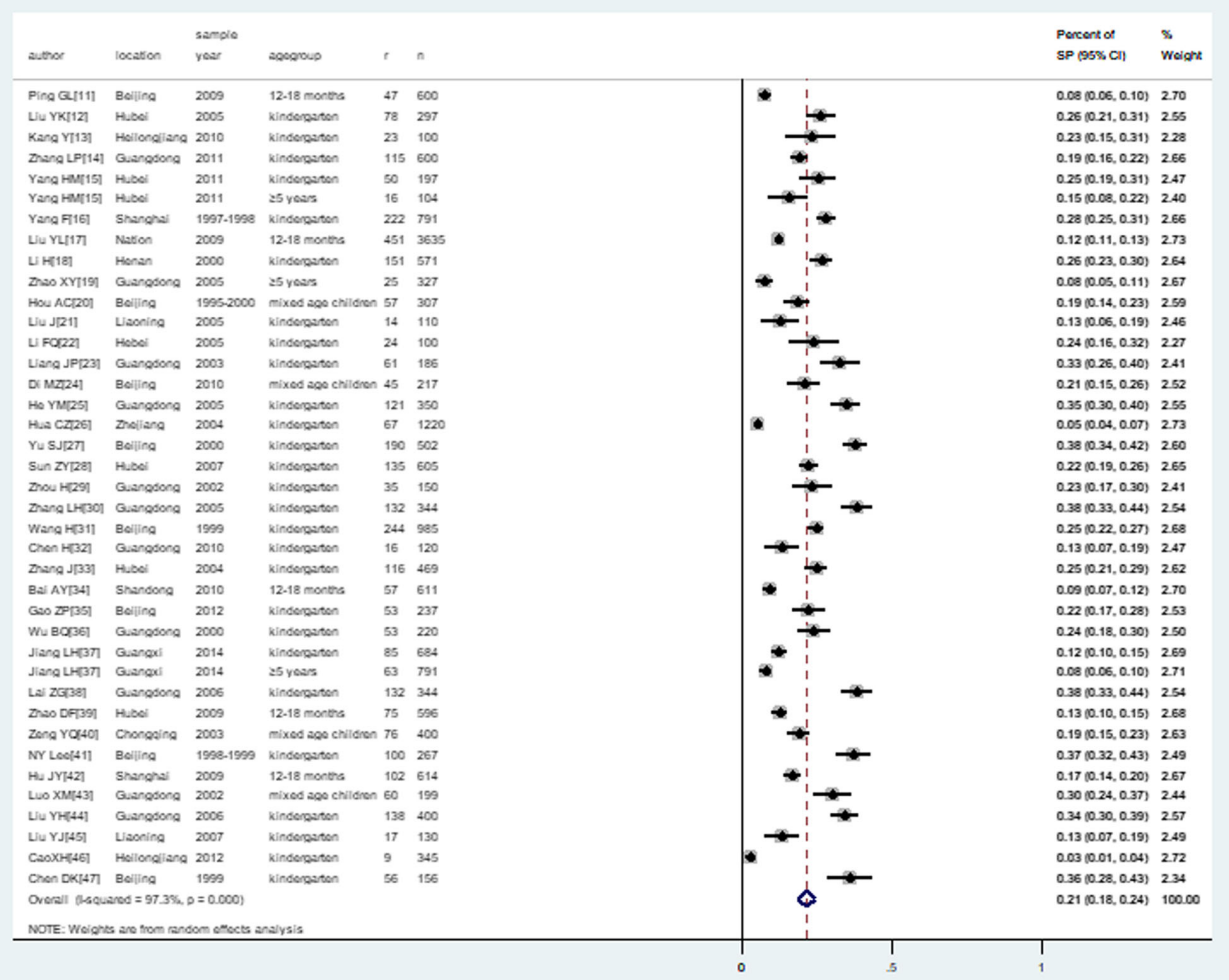
The pooled rate of nasopharyngeal carriage of *Streptococcus pneumoniae*

A total of 37 studies including 19,120 healthy children reported nasopharyngeal carriage of *S. pneumoniae.* Among them, 4 children from Di [24] and 235 children from Gao [35] reported a vaccination history, were all excluded. Finally, only 3511 colonization were reported among 18881non-vaccination children, The lowest prevalence was reported by XH Cao [46], which was 2.6% (0.9–4.3%); the highest prevalence was reported by ZG Lai [38], which was 38.4% (33.2–43.5%). The pooled prevalence of nasopharyngeal carriage of *S. pneumoniae* in healthy children was 21.4% (18.3–24.4%) (Fig. 2).

### Identification and confirmation of *S. pneumoniae* with different methods

**Fig. 3:**
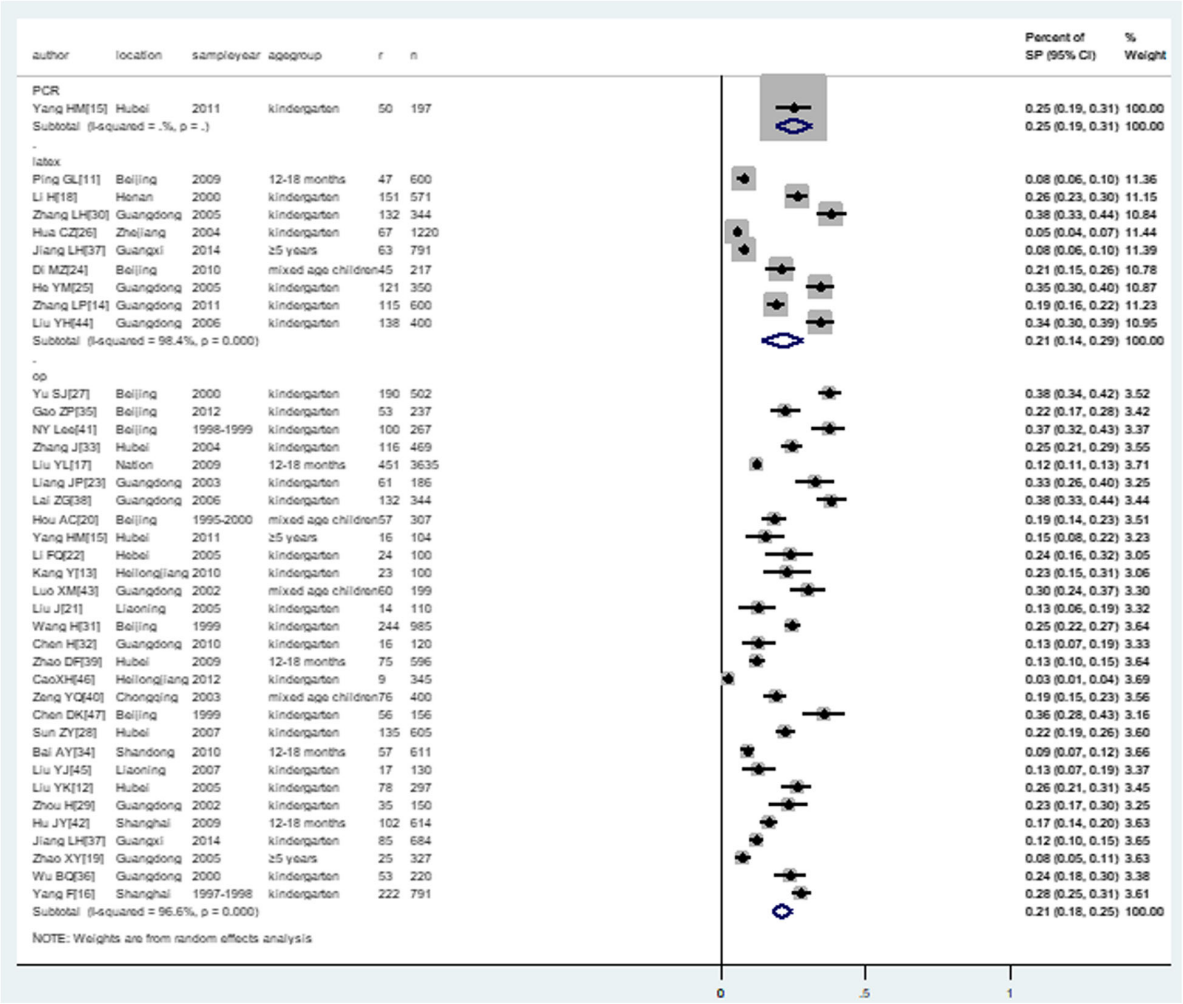
Identification and confirmation of *S. pneumoniae* with different methods

Table 2 summarizes the methods used to identify and confirm the *S. pneumoniae* strains. Three different methods, including PCR, optochin disk with bile solubility and latex agglutination were used. There was no impact on the prevalence of *S. pneumoniae* when using three different identification methods, see Fig. 3.

**Table 2:** Characteristics of sampling, culture and serotyping techniques

Author	Type of swabs	Immediately incubated into plates or not	Transportation period	Culture plates	Culture into the 5% CO_2_	Identification methods	Serotyping methods	Storage medium
Guoling Ping [11]	NP	Yes	4 h	5% sheep blood agar	Yes	Latex agglutination	N/A	Skim milk powder
Yakun Liu [12]	NP	Yes	4 h	5% sheep blood agar	Yes	Optochin disk +bile solubility	N/A	N/A
Yan Kang [13]	NP	Yes	4 h	TSA + 5% sheep blood + 5 μg/ml gentamicin	Yes	Optochin disk +bile solubility	N/A	N/A
Liping Zhang [14]	NP	Yes	4 h	5% sheep blood agar	Yes	Latex agglutination	N/A	N/A
Hongmei Yang [15]	NP	Yes	4 h	5% sheep blood agar	Yes	PCR	N/A	Skim milk powder
Fan Yang [16]	NP	Yes	4 h	TSA + 5% sheep blood + 5 μg/ml gentamicin	Yes	Optochin disk +bile solubility	Quellung	Sheep and broth
Yali Liu [17]	NP	Yes	4 h	5% sheep blood agar	Yes	Optochin disk +bile solubility	Quellung	Skim milk powder
Hao Li [18]	NP	Yes	4 h	TSA + 5% sheep blood + 5 μg/ml gentamicin	Yes	Latex agglutination	N/A	Glycerol broth
Xiyuan Zhao [19]	NP	Yes	0.5 h	TSA + 5% sheep blood + 5 μg/ml gentamicin	Yes	Optochin disk +bile solubility	N/A	N/A
Ancun Hou [20]	NP	Yes	4 h	5% sheep blood agar	Yes	Optochin disk +bile solubility	N/A	N/A
Jun Liu [21]	NP	Yes	4 h	TSA + 5% sheep blood + 5 μg/ml gentamicin	Yes	Optochin disk +bile solubility	N/A	N/A
Fuqin Li [22]	NP	Yes	4 h	TSA + 5% sheep blood + 5 μg/ml gentamicin	Yes	Optochin disk +bile solubility	N/A	N/A
Jianping Liang [23]	NP	Yes	4 h	TSA + 5% sheep blood + 5 μg/ml gentamicin	Yes	Optochin disk +bile solubility	N/A	N/A
Mingzhi Di [24]	NP	Yes	4 h	TSA + 5% sheep blood + 5 μg/ml gentamicin	Yes	Latex agglutination	N/A	N/A
Yongming He [25]	NP	Yes	4 h	TSA + 5% sheep blood + 5 μg/ml gentamicin	Yes	Latex agglutination	N/A	Skim milk powder
Chunzhen Hua [26]	NP	Nutrition broth then subculture	4 h	TSA + 5% sheep blood + 5 μg/ml gentamicin	Yes	Latex agglutination	N/A	N/A
Sangjie Yu [27]	NP	Nutrition broth then subculture	4 h	TSA + 5% sheep blood + 5 μg/ml gentamicin	Yes	Optochin disk +bile solubility	Quellung	N/A
Ziyong Sun [28]	NP	Yes	4 h	5% sheep blood agar	Yes	Optochin disk +bile solubility	Quellung	N/A
Hong Zhou [29]	NP	Yes	4 h	TSA + 5% sheep blood + 5 μg/ml gentamicin	Yes	Optochin disk +bile solubility	N/A	Skim milk powder
Lihua Zhang [30]	NP	Yes	4 h	5% sheep blood agar	Yes	Latex agglutination	N/A	N/A
Hui Wang [31]	NP	Yes	4 h	TSA + 5% sheep blood + 5 μg/ml gentamicin	Yes	Optochin disk +bile solubility	Quellung	Skim milk powder
Hui Chen [32]	NP	Yes	4 h	5% sheep blood agar	Yes	Optochin disk +bile solubility	N/A	N/A
Jing Zhang [33]	NP	Yes	4 h	5% sheep blood agar	Yes	Optochin disk +bile solubility	Quellung	N/A
Aiying Bai [34]	NP	Yes	4 h	TSA + 5% sheep blood + 5 μg/ml gentamicin	Yes	Optochin disk +bile solubility	N/A	N/A
Zhipeng Gao [35]	NP	Yes	N/A	5% sheep blood agar	Yes	Optochin disk +bile solubility	N/A	N/A
Benquan Wu [36]	NP	Yes	4 h	5% sheep blood agar	Yes	Optochin disk +bile solubility	N/A	N/A
Lihua Jiang [37]	NP	Yes	4 h	5% sheep blood agar	Yes	Optochin disk +bile solubility	N/A	N/A
Zhigang Lai [38]	NP	Yes	4 h	5% sheep blood agar	Yes	Latex agglutination	N/A	N/A
Defeng Zhao [39]	NP	Yes	4 h	5% sheep blood agar	Yes	Optochin disk +bile solubility	Quellung	N/A
Youqun Zeng [40]	NP	Yes	N/A	5% sheep blood agar	Yes	Optochin disk +bile solubility	N/A	N/A
NY Lee [41]	nasal	Yes	4 h	TSA + 5% sheep blood + 5 μg/ml gentamicin	Yes	Optochin disk +bile solubility	Quellung	N/A
Jiayu Hu [42]	NP	Yes	4 h	5% sheep blood agar	Yes	Optochin disk +bile solubility	Quellung	N/A
Xiaoming Luo [43]	NP	Yes	N/A	TSA + 5% sheep blood + 5 μg/ml gentamicin	Yes	Optochin disk +bile solubility	N/A	N/A
Yanhui Liu [44]	NP	Yes	4 h	5% sheep blood agar	Yes	Latex agglutination	N/A	Skim milk powder
Yanjie Liu [45]	NP	Yes	4 h	TSA + 5% sheep blood + 5 μg/ml gentamicin	Yes	Optochin disk +bile solubility	N/A	N/A
Xinghua Cao [46]	NP	Yes	4 h	5% sheep blood agar	Yes	Optochin disk +bile solubility	N/A	N/A
Dongke Chen [47]	NP	Yes	0.5 h	TSA + 5% sheep blood + 5 μg/ml gentamicin	Yes	Optochin disk +bile solubility	N/A	N/A

*NP* nasopharyngel swab, *TSA* Trypticase soy agar, *N/A* not mentioned or not acquired

### Nasopharyngeal carriage of *S. pneumoniae* by age

**Fig. 4:**
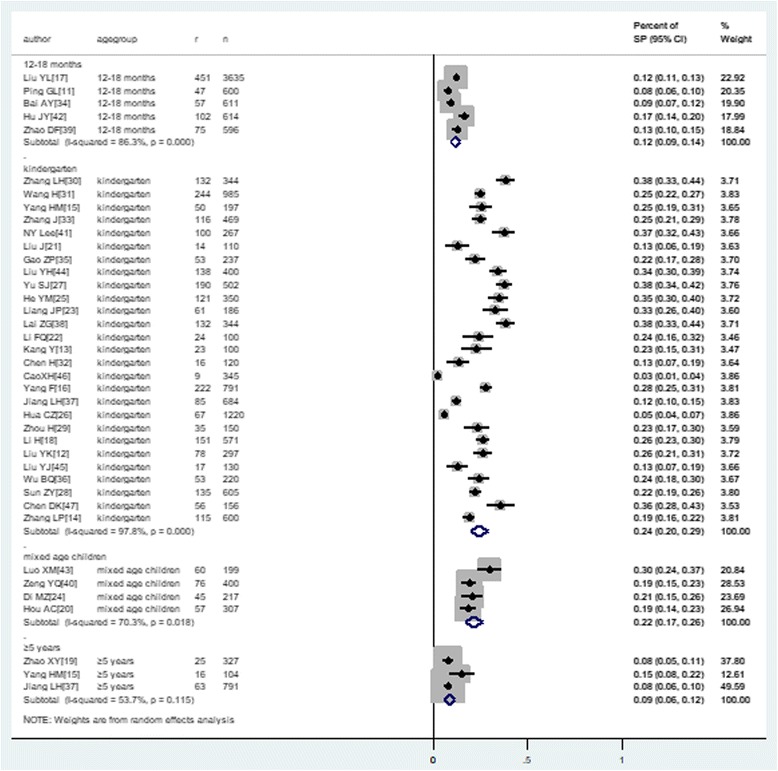
The pooled prevalence of nasopharyngeal carriage of *S. pneumoniae* distributed by age

Figure 4 summarizes the prevalence of nasopharyngeal carriage of *S. pneumoniae* in healthy children in different age groups. Six studies [11, 14, 17, 34, 39, 42] reported the prevalence of nasopharyngeal carriage of *S. pneumoniae* among children younger than 2 years of age. Among the 6656 healthy children in this age group, a total of 847 were identified to be positive for nasopharyngeal carriage of *Streptococcus pneumoniae*; thus, the pooled prevalence was 11.7% (9.1–14.2%). Twenty-seven studies [12–16, 18, 21–33, 36–38, 41, 44–47] including 10,480 kindergarten children (2–5 years of age) investigated the prevalence of nasopharyngeal carriage of *S. pneumoniae*. Within these studies, a total of 2437 children were identified to be positive for *S. pneumoniae* carriage, and the pooled prevalence was 24.5% (19.7–29.3%). Among the 1122 healthy children who were older than 5 years of age [15, 19, 37], 104 were identified as *S. pneumoniae* carriers; therefore, the prevalence of nasopharyngeal carriage was 8.8% (6.0–11.5%) in this age group. The prevalence of nasopharyngeal carriage of *S. pneumoniae* varied between the three age groups, with the highest rate reported in kindergarten children (*P* = 0.002).

### PCV7 and *S. pneumoniae* nasopharyngeal carriage

The 7-valent pneumococcal conjugate vaccine was introduced to China in October 2008, but it has not yet been included in the Chinese Expanded Program on Immunizations (EPI) [48]. Unlike the vaccination in Chinsed EPI schedule, the PCV7 vaccine was not free to the public and the coverage was estimated as 9.91% [49].

**Fig. 5:**
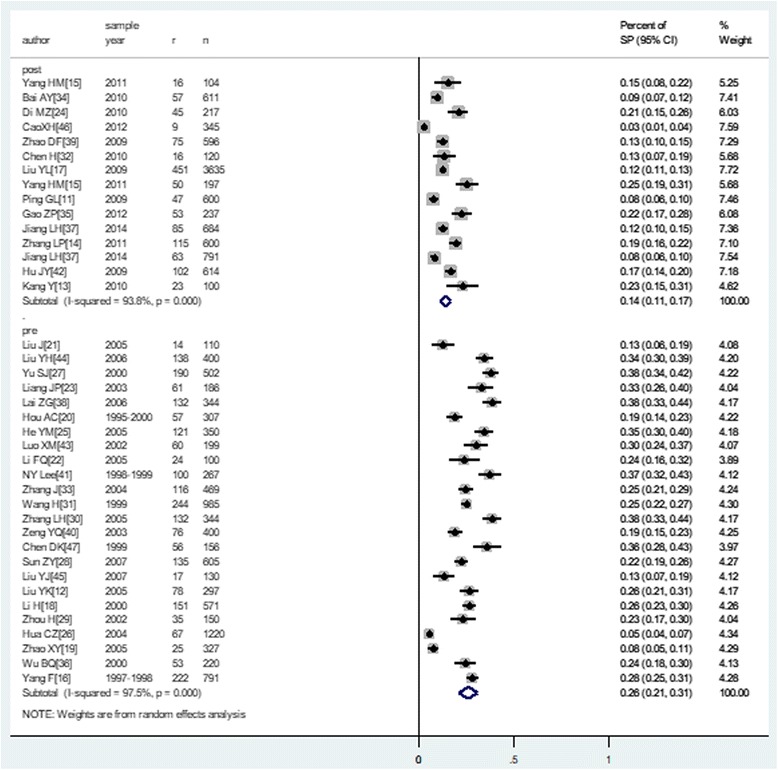
The pooled prevalence of nasopharyngeal carriage of *S. pneumoniae* stratified by vaccination period

Before the PCV7 was introduced in mainland China, 24 studies [12, 16, 18–23, 25–31, 33, 36, 40, 41, 43–45, 47] had reported the prevalence of nasopharyngeal carriage of *S. pneumoniae*; within these studies, the pooled prevalence was 25.8% (20.7–30.9%), Fig. 5. The prevalence of nasopharyngeal carriage sharply declined following the introduction of PCV7, with a pooled prevalence of 14.1% (11.3–16.9%) identified in studies conducted post-PCV7 introduction [11, 13–15, 17, 24, 32, 34, 35, 37, 39, 42, 46]. There was a highly significance differences in the prevalence between these two time periods (*P* < 0.001). In kindergarten children, before the pcv7 vaccination period, the pooled prevalence was 27.2% (21.3, 33.2%) and 16.6% (9.5, 23.7%) in the post vaccination period (*P* < 0.001).

### Overall heterogeneity and publication bias

Stratified analyses were carried out to assess the heterogeneity across subgroups defined by age, PCV7 introduction period and PCV7 introduction period within kindergarten children groups. The sensitivity analysis indicated that the pooled prevalence of *S. pneumoniae* carriage had only slight variations by stratified studies into pre/post vaccination period when individual studies were omitted one by one. The prevalence estimates ranged from 13.4% (10.6, 16.1%) to 14.8% (12.5, 17.1%) in post vaccination period and from 25.2% (20.7, 31.1%) to 26.3% (21.1, 31.6%) in pre-vaccination period, suggesting that the results were stable.

Slight publication bias was noted from the statistical tests (Egger’s test, *P* = 0.011; Begg’s test, *P* = 0.01). After stratified the pooled prevalence of *S. pneumoniae* by PCV7 vaccination period, the potential publication bias was adjusted as no significant (Egger’s test, *P* = 0.134; Begg’s test, *P* = 0.602) in pre-vaccination period and (Egger’s test, *P* = 0.353; Begg’s test, *P* = 0.125) in post vaccination period.

### Antibiotic resistance profiles of the isolates

A total of 20 studies [11, 12, 15, 16, 18, 20, 21, 23, 25–28, 31, 33, 34, 36, 37, 40–42] were identified that reported antibiotic resistance in *S. pneumoniae*. The rate of pneumococcal resistant to levofloxacin was 2.5% (0.3–4.6%), which was the lowest rate of antibiotic resistance identified. The highest resistant rate was reported against tetracycline antibiotics; for this class of antibiotics, a pooled resistance rate of 67.1% (33.8–96.4%) was identified. The pneumococcal resistance rate to penicillin was 28.9% (20.4–37.4%). Before the introduction of PCV7 [12, 16, 18, 20, 23, 25–28, 31, 33, 36, 40, 41], the pooled resistant rate to penicillin was 31.9% (21.2–42.6%). This rate decreased by 21.6% (7.4–35.9%) following the introduction of PCV7 [11, 15, 21, 34, 37, 42]. The penicillin resistant rate varied significantly between the pre- and post-PCV7 time periods (*P* < 0.001) (Table 3). The results of subgroup analysis indicated that the heterogeneity of resistant to penicillin may came from pre/post vaccination period, while the rest of them may came from different age groups. A slightly publication bias was found in Chloromycetin resistant rate, no publication bias was found in the rest of the antibiotics.

**Table 3:** The resistance of antibiotic among all the *S. pneumoniae*

Antibiotic	No. of studies	Total no. of included strains	No. of included strains with antibiotic resistant	Resistant rate(%) (95%CI)	*I* ^*2*^	*P*	*P* value of Egger’s test	*P* value of Egger’s test
Penicillin	20	2105	541	28.9(20.4, 37.4)	69.9	0.000	0.147	0.298
Cefaclor	6	499	463	65.8(51.2, 80.4)	91.6	0.000	0.434	0.462
Ceftriaxone	8	771	90	19.4(9.2, 29.5)	96.9	0.000	0.175	0.266
Levofloxacin	13	1175	159	2.5(0.3, 4.6)	70.2	0.009	0.226	0.602
Erythromycin	14	1635	1185	65.9(57.0, 74.9)	93.6	0.000	0.131	0.108
Clindamycin	9	878	675	64.0(45.5, 82.5)	96.2	0.000	0.247	0.221
Tetracycline	12	1334	967	67.1(33.8,96.4)	99.7	0.000	0.249	0.548
Cotrimoxazole	13	1524	1103	64.5(51.2, 77.8)	96.7	0.000	1.000	0.704
Chloromycetin	13	1524	360	24.1(16.7, 31.5)	91.8	0.000	0.039	0.019

### Serotypes and *S. pneumoniae* nasopharyngeal carriage

**Fig. 6:**
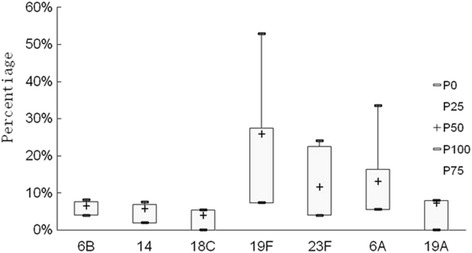
The pooled prevalence of major serotypes of *S. pneumoniae*distributed among healthy children in China

Nine studies [17, 18, 29, 30, 33, 35, 41, 43, 44] reported the serotypes of *Streptococcus pneumoniae*. In the 1626 isolates evaluated, 11 different serotypes were identified, and the predominant serotype was 19F. The pooled prevalence of serotype 19F was 19.1% (12.2–26.0%). The least prevalent serotype was 18C, which was identified in 3.2% (0.1–6.3%) of isolates (Fig. 6, Table 4). Of the 1626 isolates, 755 were identified as serotypes included in the coverage of PCV7, and 1059 were identified as serotypes included in the coverage of PCV13. The serotype coverage rates were 46.6% (38.8–54.4%) for PCV7 and 66.2% (58.6–73.8%) for PCV13. >Before PCV7 was introduced in mainland China [16, 27, 28, 31, 38, 41], the serotype coverage rates of PCV7 and PCV13 were 43.9% (34.1–53.6%) and 66.8% (56.1–76.0%), respectively. These rates changed to 52.1% (37.3–66.9%) and 66.3% (50.6–81.9%) for PCV7 and PCV13, respectively, following the introduction of PCV7 [17, 39, 42].

**Table 4:** Analysis of major serotypes of *Streptococcus pneumoniae*

Serotype	No. of studies	Total no. of included strains	No. of included strains with identical serotypes	Prevalence(%)(95%CI)	*I* ^*2*^	*P*	*P* value of Egger’s test	*P* value of Egger’s test
23F	9	1175	187	14.0(8.4–19.7)	89.4	0.000	0.266	0.193
6A	9	1175	174	11.9(6.3–17.5)	90.2	0.000	0.032	0.063
19F	9	1626	322	19.1(12.2–26.0)	93.3	0.000	0.754	0.602
6B	6	1175	86	6.8(4.7, 8.9)	0.0	0.474	0.739	0.902
14	8	1382	85	5.5(4.0, 6.9)	31.3	0.187	0.910	0.754
18C	3	397	16	3.2(0.1, 6.3)	68.6	0.041	0.631	0.620
15	7	1332	86	5.7(3.6, 7.8)	63.6	0.011	0.502	0.548
19A	4	1192	99	8.7(5.9–11.6)	65.6	0.008	0.142	0.764
PCV7		1626	755		90.2	0.000	0.953	0.917
PCV13		1626	1059		90.0	0.000	0.644	0.602

Heterogeneity was detected in the serotype distributions of 23F, 6A, 19F, 18C, 15, 19A and PCV7, PCV13 vaccine coverage rate (all *P* values were <0.05), although after sequential exclusion of each study, the conclusion was not affected by the exclusion of any specific study.

## Discussion

This systematic review analyzed the prevalence and serotype distributions of nasopharyngeal carriage of *S. pneumoniae*, antibiotic resistant rates in *S. pneumoniae*, and the rates corresponding the serotype coverage provided by PCV7 and PCV13.

Since the serotypes distribution of and antibiotic resistance in *S. pneumoniae* isolates have been found to vary from region to region, the prevalence of *S. pneumoniae* has also been found to vary in different populations. The prevalence of nasopharyngeal carriage of *S. pneumoniae* was found to be 60% in infants under 2 years of age in Greenland [49], while the prevalence of nasal carriage was only identified as 9.8% in elderly populations in Italy [50]. In Hong Kong, the prevalence of nasopharyngeal carriage *S. pneumoniae* was identified as 13.5% in children younger than 5 years of age who had never received any pneumococcal vaccines, 14.1% in children who received at least one dose of PCV13, and 15.3% in children who received at least 3 doses of the PCV13 vaccine [51]. In Taiwan, the prevalence of nasopharyngeal carriage of *S. pneumoniae* identified in children younger than 5 years of age was 14.1%, similar to that identified Hong Kong [52]. However, data collected in mainland China have differed from data collected in Taiwan and Hong Kong. The pooled prevalence of nasopharyngeal carriage of *S. pneumoniae* was determined to be 21.4% (18.3–24.4%) among children in China.

A variety of studies have confirmed that colonization by *S. pneumoniae* begins in infanthood and early childhood. It has been reported that carriage of this pathogen is acquired within the first 6 months of life and, the prevalence of the epidemic appeared to peak in children of pre-school age [53]. A study conducted by Ueno M [53] showed that prevalence of nasopharyngeal carriage of *S. pneumoniae* increased with age within pediatric age groups, with rates of 19 and 23% identified in infants younger than 1 years-old and children 2 to 3 years old, respectively. The highest prevalence has been identified during the pre-school period. Our data were consistent with the findings of Ueno M [53], suggesting that carriage trends differed with age. The prevalence was 12.8% (10.0–15.6%) in children younger than 2 years old; the prevalence increased with age and reached a peak at 24.7% (19.7–29.7%) in children aged 2 to 5 years and then decreased to 8.8% (6.0–11.5%) in children aged 5 years and older. It is well known that attending kindergarten has been identified as a risk factor [52, 53] for colonization by opportunistic pathogens, such as *S. pneumoniae,* due to poor hygiene, confined physical environmental conditions and frequent interaction with other children. Nasopharyngeal carriage of *S. pneumoniae* in kindergarten children results in this population serving as an asymptomatic reservoir that spreads this pathogen into community. Since the PCV7 was introduced into China in October 2008, the studies conducted between 2009 to 2012 in age 2 to 5 years-old children were the coverage and the active population of getting shot by PCV7 vaccine, which leads to a reduction of prevalence of nasopharyngeal carriage of *S. pneumoniae*.

Unlike the GAVI Alliance [54] in the world and EPI in China, the PCV7 is available at immunization clinics for a fee during 2008–2015, these clinics designated as “point of vaccination” centers, children at 2, 4, 6 months will get shot of one dose of PCV7 and at 1 years old will get the fourth shot of does to enhance the immunity after purchase the vaccine [54]. Because of the high price of PCV7, the PCV7 coverage level was not as many other countries [8, 9]. According to a survey of children age 1 to 2 years selected from 31 provinces throughout China conducted in 2012, 9.9% of children had received one dose of PCV7 [49]. Another study from Shanghai reported a similar PCV7 coverage level at 11.4% [55]. We observed a slightly change of PCV7 coverage level from 43.9% (34.1, 53.6%) to 52.1% (37.3, 66.9%) between pre/post vaccination period because of the limited herd immunity from low vaccine rate of pneumococcal conjugate vaccination.

High antibiotic resistance rates in *S. pneumoniae* may facilitate transmission of this pathogen among young children. Crowding and barriers to maintaining quality hygiene facilities could accelerate the transmission of highly antibiotic resistant *S. pneumoniae* in the kindergarten environment [56]. Our pooled data indicated that the rates of erythromycin, clindamycin, trimethoprim- sulfamethoxazole and tetracycline resistance among isolates were all more than 60%. High-level resistance to the aforementioned antibiotics has also been identified in previous studies [57]. Macrolides and lincosamides have been reported to be the first-line empirical antibiotic therapy for pneumococcal infections in China, and the use of these agents has led to a high rate of antibiotic resistance in *S. pneumoniae* [42, 57]. Previous studies have demonstrated that the penicillin-non-susceptible pneumococci (PNSP) rate varied in different regions. The prevalence of nasopharyngeal carriage of *S. pneumoniae* in Brazilian and Korean children who attended day care centers were identified as 26.0 and 31.3%, respectively [58, 59]. A marked modification in pneumococcal antibiotic susceptibility rates was observed after the introduction of pneumococcal conjugate vaccines. The PNSP rate was 47.1% before the introduction of PCV13 in France, and this rate rapidly decreased to 39% 3 years after PCV13 was introduced [60]. The pooled data in this study were consistent with results identified in France. The proportion of pneumococcal isolates resistant to penicillin identified in this study decreased from 31.9% (21.2–42.6%) to 21.6% (7.4–35.9%) after the introduction of PCV7.

A remarkable decrease in the incidence and mortality of invasive pneumococcal disease has been observed following the introduction of pneumococcal conjugate vaccines into pediatric immunization programs [61]. With the introduction of these PCVs and further reductions in the prevalence of nasopharyngeal carriage of *S. pneumoniae* in pediatric groups. Our data demonstrated that the prevalence of nasopharyngeal carriage of *S. pneumoniae* was 25.8% (20.7–30.9%) among healthy children before the introduction of PCV7. The prevalence dropped sharply to 14.1% (11.3–16.9%) following the introduction of PCV7 in China, indicating that the impact of PCV7 introduction on disease prevalence can be determined by assessing the nasopharyngeal carriage of *S. pneumoniae* in healthy children.

## Conclusions

Pneumococcal carriage was identified to occur at generally high prevalence among children in China. PCV7 immunization was associated with a reduction in the rate of penicillin resistance among nasopharyngeal carriage isolates of *S. pneumoniae*. The distribution of serotypes identified in the nasopharynx was only slightly modified following the introduction of the PCV7 vaccination because of the low PCV7 immunization rates. The Centers for Disease Control and Prevention should timely adjust PCV vaccination strategies based on these findings to reduce the incidence and morbidity of pneumococcal invasive disease in pediatric populations.

## Additional file

Additional file 1: Data base. (XLSX 25 kb)

## Abbreviations

CIs: Confidence intervals
IPD: Invasive pneumococcal disease
NVT: Non-vaccine type
PCV: Pneumococcal conjugate vaccine
PNSP: Penicillin-non-susceptible pneumococci
REM: Random-effects model
*S. pneumonia*: *Streptococcus pneumonia*
VT: Vaccine type
